# Handheld Lasers Allow Efficient Detection of Fluorescent Marked Organisms in the Field

**DOI:** 10.1371/journal.pone.0129175

**Published:** 2015-06-02

**Authors:** Kevin B. Rice, Shelby J. Fleischer, Consuelo M. De Moraes, Mark C. Mescher, John F. Tooker, Moshe Gish

**Affiliations:** 1 Department of Entomology, The Pennsylvania State University, University Park, PA, United States of America; 2 Department of Environmental Systems Science, ETH Zürich, Zürich, Switzerland; AntiCancer Inc., UNITED STATES

## Abstract

Marking organisms with fluorescent dyes and powders is a common technique used in ecological field studies that monitor movement of organisms to examine life history traits, behaviors, and population dynamics. External fluorescent marking is relatively inexpensive and can be readily employed to quickly mark large numbers of individuals; however, the ability to detect marked organisms in the field at night has been hampered by the limited detection distances provided by portable fluorescent ultraviolet lamps. In recent years, significant advances in LED lamp and laser technology have led to development of powerful, low-cost ultraviolet light sources. In this study, we evaluate the potential of these new technologies to improve detection of fluorescent-marked organisms in the field and to create new possibilities for tracking marked organisms in visually challenging environments such as tree canopies and aquatic habitats. Using handheld lasers, we document a method that provides a fivefold increase in detection distance over previously available technologies. This method allows easy scouting of tree canopies (from the ground), as well as shallow aquatic systems. This novel detection method for fluorescent-marked organisms thus promises to significantly enhance the use of fluorescent marking as a non-destructive technique for tracking organisms in natural environments, facilitating field studies that aim to document otherwise inaccessible aspects of the movement, behavior, and population dynamics of study organisms, including species with significant economic impacts or relevance for ecology and human health.

## Introduction

Field studies that track or recapture marked organisms can provide key insights into otherwise inaccessible aspects of life histories and behaviors of study organisms. Furthermore, such methods can be used to study movement or local population dynamics of ecologically and economically important organisms, including endangered or invasive species, agricultural pests, and disease vectors. A number of different marking and detection techniques have been developed to facilitate such studies, and available methods vary greatly in their efficiency (e.g. mark retention and recapture rates), logistical complexity and cost, scalability (for studies with large populations), and potential impacts on marked organisms [1]. Fluorescent pigments provide one of the most efficient and cost-effective marking techniques, but limitations on the efficacy with which such marks can be readily detected in the field (especially over distances greater than a few meters) present a significant drawback to their use.

Ecologists have been using fluorescent powders and dyes for more than 65 years to investigate the behavior and movement of birds, reptiles, mammals, fish, mollusks, insects, and other animals [2–9]. Fluorescent markings are typically visible in daylight and glow intensely under ultraviolet (UV) light, due to conversion of radiant energy from UV light sources to longer wavelengths visible to the human eye [10]. However, because fluorescent UV lamps typically enable detection at distances of only a few meters, their use in the field can be time and labor intensive, and studies using fluorescent pigments often rely instead on re-capture of organisms which are then returned to the laboratory and examined for markings under UV light [11–14]. However, despite requiring repeated handling of study organisms for marking and detection, use of fluorescent pigments can be quick and cheap, particularly when compared to newer and more expensive techniques such as protein marking and harmonic radars [1, 15–17]. Furthermore, while some alternative tracking techniques can avoid issues associated with the need to initially handle organisms for marking, these techniques- including the use of elemental, isotopic, or genetic markers- can also be relatively expensive, time-consuming, and technically complex [18–21].

Development of effective methods for detecting fluorescent pigments at greater distances in the field, would greatly enhance usefulness of such markers in non-destructive studies that involve repeated observations of marked individuals in their natural habitats without (or prior to) recapture and handling. Recent availability of UV light-emitting diode (LED) flashlights has increased potential re-sight distances compared to those provided by portable fluorescent UV lamps, but recent advances in LED technology have also led to the development of powerful UV-LED lasers that are increasingly affordable and available. To evaluate the potential value of these newly available UV sources for ecological field research, the current study compared re-sight distances and detection efficiency of fluorescent-marked insects in the field for UV xenon lights, UV-LED flashlights, and powerful handheld near-UV lasers (405nm) equipped with a focusing lens (and used while wearing specially designed protective eyewear). The goal of the study was to identify novel methods capable of increasing re-sight distances and detection efficiency for marked organisms and, consequently, of improving the efficacy of mark-recapture studies and potentially allowing use of such studies in habitats, such as dense forest canopies, where they have previously had limited application.

## Materials and Methods

We tested the feasibility of four different light sources for nighttime detection of fluorescent-marked organisms: (1) UV LED medium-beam flashlight (3-watt UltraFire WF-502B, 365nm, no filter, Arizona Tactical Gear, Phoenix, AZ, $30, 3.7 volt battery); (2) UV LED narrow-beam spotlight (UVG3 Spotlight Torch, 365nm, visible light filter, Labino, Solna, Sweden, $660, 3.7 volt battery); (3) UV xenon floodlight (50-watt SuperXenon Midlight, peak-365nm, visible light filter, Labino, Solna, Sweden, $2,000); (4) handheld focusable laser (PX 600mW [500mW average], class IIIB purple laser, 405nm, Big Lasers, New York, New York, $260, 3.7 volt batteries). The effective power of the laser was 580 mW measured with an Ophir power meter (3A-P-SH-V1-ROHS) and Ophir Nova Display (ROHS) (Ophir Optronic Solutions, Jerusalem, Israel).

### Target species and marking

As a model study organism, we used fluorescent-marked brown marmorated stink bugs (BMSB), *Halyomorpha halys* Stål, an invasive pest species that is active across field and forest edges [22–23]. Freshly killed BMSB were placed in a 16 × 15-cm plastic bag with 1 g of red fluorescent powder (BioQuip, Rancho Dominguez, CA) and gently shaken for five seconds. Marked insects were then used in detection assays as described below. Brown marmorated stink bugs were collected from university land and no ethics or collecting permits were required.

### Detection distance under field conditions

For this assay five fluorescent-marked BMSB were glued 2-cm apart in a horizontal row on a black, felt-covered, 30 x 50 cm cardboard sheet that was staked 0.5 m above ground level in an open field. We placed measuring tape on the ground to record distance from the backboard. For each light source, seven inexperienced volunteers were asked to aim the light at the target after dark (between 8:00PM-12:00AM) and walk backwards until they reached their maximum detection distance for the marked insects (the last distance they subjectively felt would be a reliable working distance if they were scouting BMSB in the field). While volunteers were walking away from the board, they were facing a half moon, simulating non-ideal lighting conditions that are often encountered in field studies. The focus of the laser produced a 1-m diameter beam at a distance of 40 m. We used AVOVA to compare light-source detection distance followed by Tukey’s HSD means separation test. While working with UV light sources, volunteers wore clear UV-filtering goggles. While working with the laser, volunteers wore opaque protective goggles with a clear round 2.5-cm diameter window fitted with an EdgeBasic long-wave pass filter (transmission band: 454–900nm, Semrock Inc., Rochester, NY, $350). The filter eliminates purple light from the laser but does not interfere with transmission of the rest of the visible light spectrum, allowing convenient detection of all fluorescent colors except purple (there is also a slight reduction in blue fluorescence). A researcher using this method may install, instead of the 2.5-cm diameter filter we used, a larger filter that would make scouting more convenient.

### Detection efficiency under field conditions

For this assay, we haphazardly placed twenty marked BMSB in the leafy canopy of a 27-m high oak tree (with a canopy diameter of 28 m). Marked BMSB were glued to fishing line and tied to tree foliage. The height of marked bugs in the tree was calculated using a TruPulse 200 laser range finder (Laser technology Inc., Centennial, CO). When placed in the tree canopy, the marked BMSB were not visible from the ground during the day. We conducted this experiment with the laser because it had the greatest detection distance (see Results) and with the xenon lamp because it had the widest beam. Nine volunteers (with no prior knowledge of the location of the targets) scouted individually with the laser and two scouted with the xenon lamp. Volunteers could adjust the focus of the laser to their preference. We instructed volunteers to record the number of BMSB they found and stop when they could no longer detect additional BMSB (or after a maximum scouting time of 20 minutes). To minimize the effect of battery drain on detection efficiency, each volunteer received fully charged batteries for the laser, both for the tree scouting and for the distance measurements.

### Detection of fluorescent markings in an aquatic environment

We tested the ability of the laser and UV spotlight to detect fluorescent-marked targets submerged in water. The UV LED medium-beam flashlight and UV xenon floodlight did not fluoresce submerged targets and therefore were not used in this experiment. We coated 1-cm^2^ squares of thin black plastic with three colors of fluorescent powder (red, blue, and yellow; BioQuip, Rancho Domiguez, CA), placed them between two layers of clear packing tape, and glued them to a clear plastic backboard. We submerged the backboard at a depth of two m in an indoor swimming pool and placed measuring tape along the side of the pool to record distance. Lights in the facility were turned off to simulate night conditions. We instructed ten volunteers to walk away from the submerged targets, along the pool edge, until they reached a reliable working distance if they were scouting fluorescent organisms in the field. Although clearer than natural bodies of water, this simulated water-resistant tagging, e.g. visible implant fluorescent elastomer tags [24–25] and other types of aquatic tags [26] that could be made from fluorescent materials. We used AVOVA to compare detection distance between the laser and UV spotlight.

## Results

**Fig 1:**
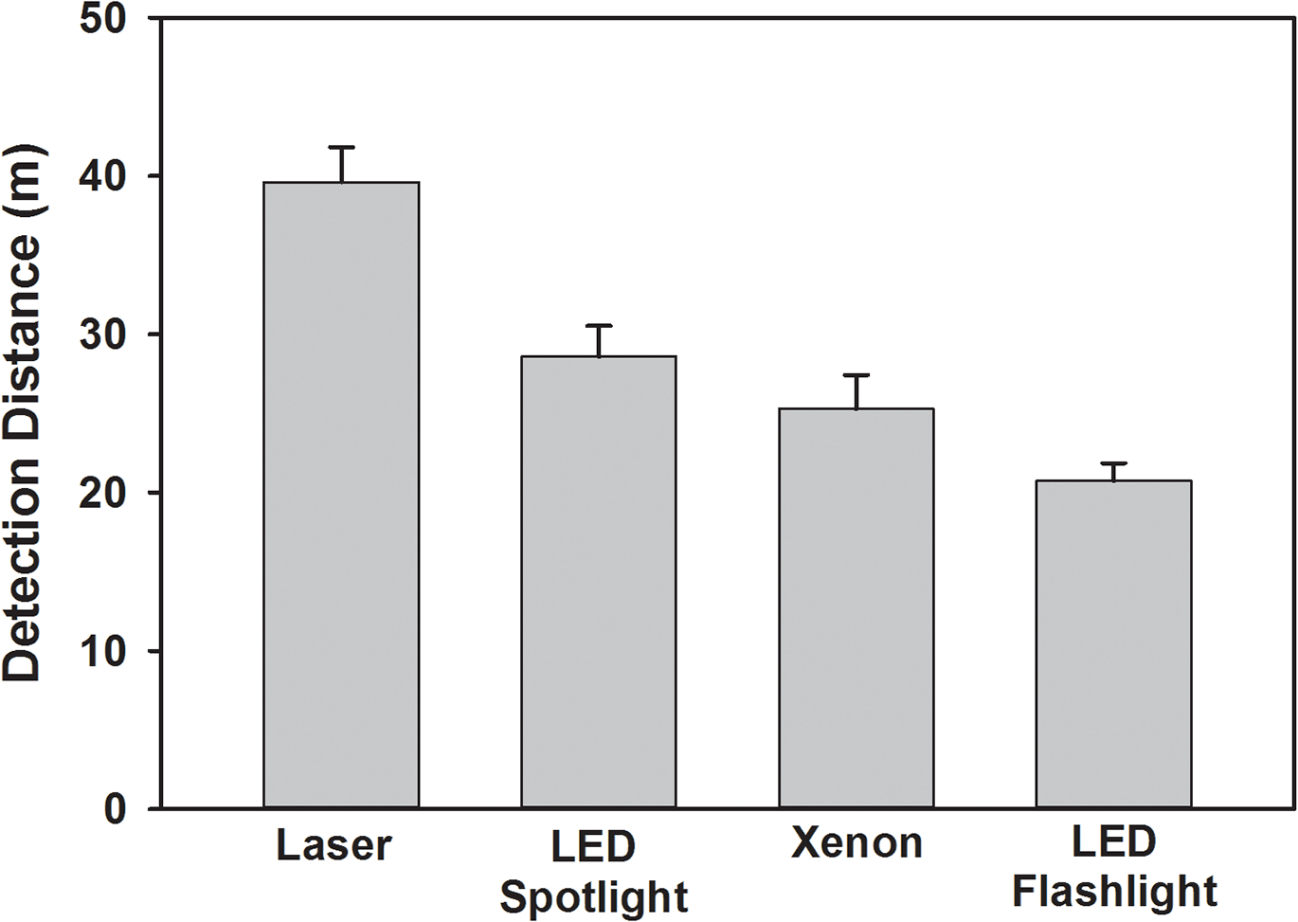
Detection distance of fluorescent-marked brown marmorated stink bugs using different ultraviolet and near-ultraviolet lights.

**Fig 2:**
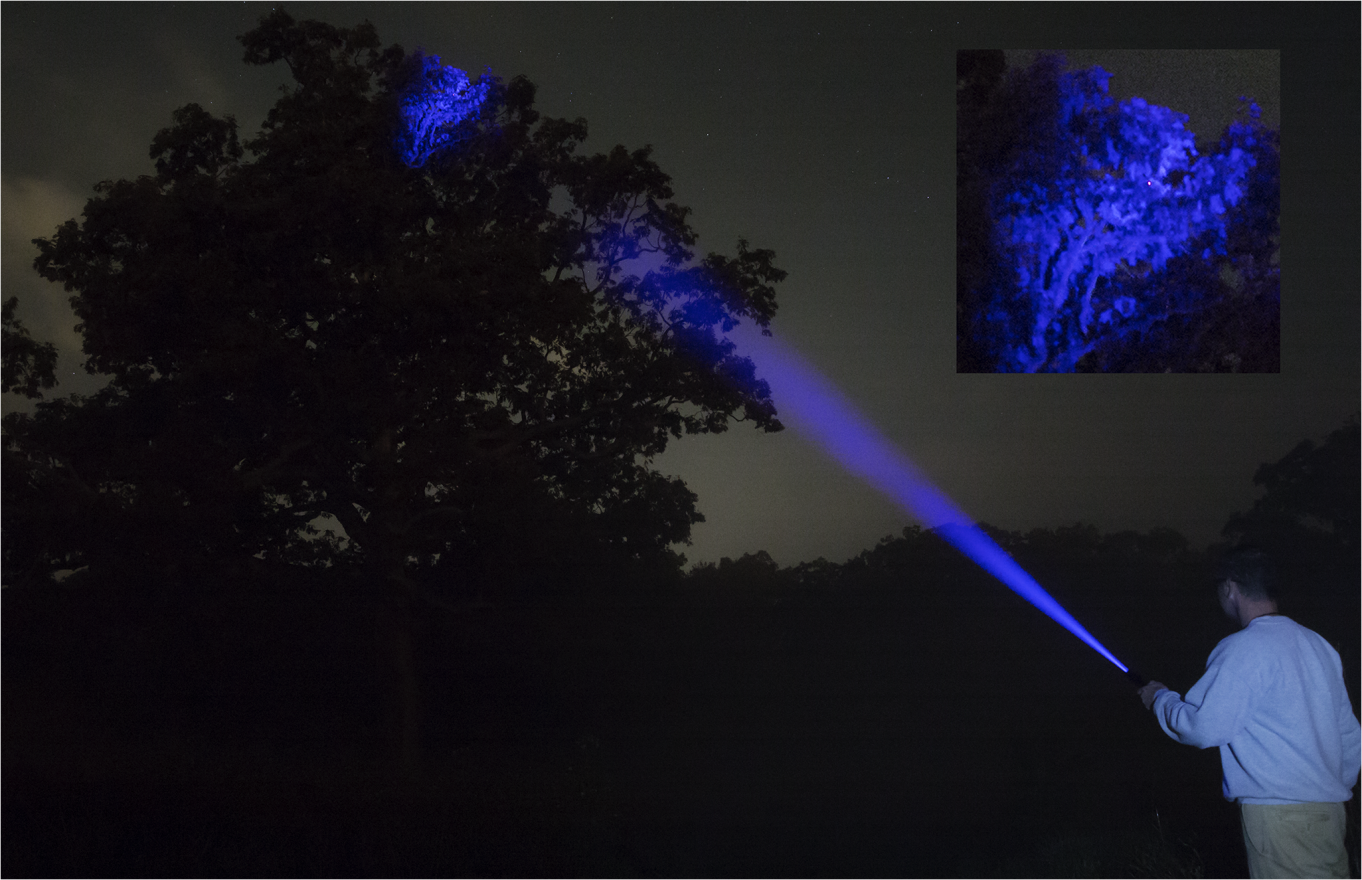
A fluorescent-marked brown marmorated stink bug detected using handheld laser equipped with a focusing lens. The oak tree in the picture is 27-m high. The specially designed goggles that we used eliminated the purple light of the laser (seen blue in the picture) and transmitted, with no interference, the light from the glowing, marked insect. The inset shows the marked insect (in red), which is clearly visible to the laser operator.

**Fig 3:**
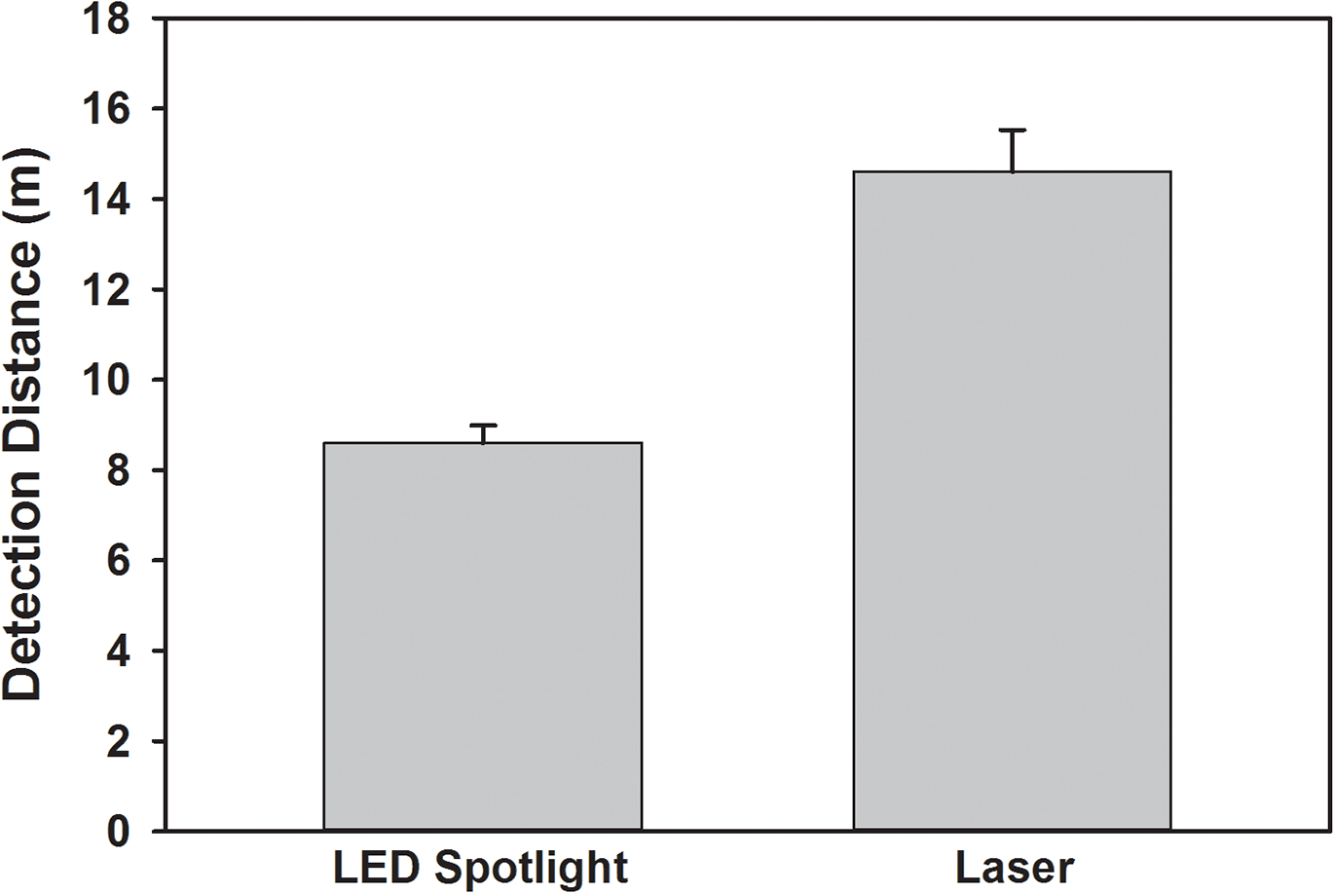
Detection distance of fluorescent tags submerged 2m in water using narrow beam ultraviolet LED spotlight and handheld laser.

All four light sources elicited fluorescence from the marking powders. The laser provided the greatest detection distance with a mean of 39.6 ± 2.2 m, approximately 10–20 m farther than the LED or xenon light sources (Fig 1; *F*_3_,_9_ = 15, *P* < 0.0001). The narrow-beam spotlight had a greater detection distance than LED flashlights (Tukey’s post hoc test *P* < 0.05). In the detection efficiency test, the laser allowed easy scanning of the top of the tree canopy (Fig 2). Scanning a tree with full foliage, volunteers using the laser detected 80 ± 0.7% of BMSB with an average scouting time of 14 min. Volunteers with the xenon lamp did not detect the five BMSB placed at the top of the canopy (above 20 m), but within 20 minutes they did detect 77± 10% of the 15 BMSB that were placed lower in the canopy. The laser detected submerge fluorescent tags in the pool at a greater distance with a mean of 14.6 ±0.93 m approximately 6 m farther than the UV spotlight 8.59 ± 0.39 m (Fig 3; *F*_1_, _10_ = 35.4, *P* < 0.0001).

## Discussion

As noted above, fluorescent marking provides a relatively inexpensive and easy-to-use method for tracking study organisms in the field; however, short re-sight distances, which have remained under four meters for over 65 years [11–14, 27], significantly limit the ability of investigators to effectively detected marked individuals within large search areas. For instance, a 200-m^2^ ground area required approximately 28 person-hours to thoroughly search [27], and marked individuals may frequently disperse from the detection range of investigators [12]. Introduction of UV LED technology increased re-sight distances, enabling detection of 5-cm long winged tree seeds (samaras) from a distance of eight meters [28]. Our results with newer LED flashlights and a handheld laser document a far larger detection distance; indeed, with the laser we achieved over a five-fold greater detection distance than any previous handheld detection technology.

The focusing lens of our laser produced a 1-m wide field that was large enough to quickly scan entire trees or local forest canopies. Furthermore, once volunteers in our study detected a marked BMSB, focusing the laser permitted clear and easy verification of the spotting. Focusing the laser to a narrower beam while scanning would further increase the detection distance (beyond 40 m), but would increase the time needed to cover a given focal area.

Availability of more powerful UV lights, and especially focusable lasers, greatly enhances the feasibility of tracking marked organisms in trees and forest canopies. Using the laser, our volunteers detected, on average, 80% of marked BMSB within 14 min, and easily detected marked insects placed near the top of a 27m tree with full foliage. Although marked insects on the upper side of foliage or branches may not be easily detected by a light beam directed from below, the higher detection distance provided by the laser allowed our volunteers to move around and away from the tree to scan its canopy from different angles, overcoming visibility problems associated with looking up through the canopy. It is also worth noting that the relatively high detection distance coupled with the low and decreasing cost of the laser (currently ~$260) and the ease of marking with fluorescent powders allows the potential of marking large numbers of organisms, which could further enhance the usefulness of such methods in areas with dense foliage or vegetation.

The laser detection method also proved effective for re-sighting in water. The laser provided 75% greater detection distance compared to the narrow-beam LED spotlight. The intensity of the laser may therefore significantly improve detection distances in aquatic studies that make use of fluorescent tags.

Deployment of such improved detection techniques is certain to increase the percentage of marked organisms found and reduce searching time, increasing the attractiveness of non-destructive field sampling via fluorescent markers relative to more expensive, laboratory-based detection techniques that require recapture and handling of study organisms. Harmonic radar also provides portable non-destructive sampling of arthropods. However, each organism must be individually tagged with a transponder, thus preventing mass marking that can be achieved with fluorescent powders [16]. The cost of harmonic radar (~$7,000) and required training [29] is much greater than the laser. The laser provides four-fold increased detection distance compared to previous studies using harmonic radar to detect insects [29]. The use of efficient handheld lasers for rapid, non-destructive detection of fluorescently marked targets may positively affect other disciplines where fluoresce detection is used, such as cancer research [30].

### Choice of marking color

It is important to note that intense UV or near-UV light projected at night has a potential of influencing animal behavior; therefore, researchers should consider this possibility and test for such effects prior to committing to LED or laser-based methods. The choice of marker color can also influence the potential for such effects, as well as for the efficacy of detection.

The human eye has greatest sensitivity in the green region of the visual spectrum [31]; consequently, markers that fluoresce green would capitalize on the spectral sensitivity of human vision. Although we did not test performance of different marking colors for their contrast with green background foliage, we do not expect this contrast to be a significant issue at night: the light sources that we tested (especially the laser, although not the unfiltered medium-beam flashlight) emit only very low amounts of white light, making it likely that green-light reflectance from foliage will be negligible. The laser that we used also induces faint chlorophyll fluorescence (Kautsky effect) [32, 33], which causes some leaves to appear red when viewed from a close distance. However, this fluorescence was unnoticeable compared with the strong fluorescence of the red marking powder, and it did not affect searching efficiency. One potential drawback of using green or yellow markers is that some arthropods naturally fluoresce green or yellow. Natural fluorescence of non-target organisms will differ among locations, and should be taken into account before choosing a marking color.

The fluorescent markings employed in our field tests were red, a color to which the human eye is relatively insensitive. We chose red to provide the most conservative detection-distance measurements, taking into account that researchers will often need to use several different colors in the same study. If using multiple colors, a researcher should test and consider the detection distance of each marking color, because differences in eye sensitivity and fluorescence intensity could influence recovery rates. If scouting for marked organisms that are located underwater, differences in color absorption by water should also be considered when the reflected light has to travel through more than a few meters in the water.

The prototype safety goggles used in this study included a type of filter typically used in fluorescence microscopy. When used in the goggles, this filter mimicked performance of conventional dark-red safety goggles, with a high optical density of six, even when the laser hits on an angle. When looking through these safety goggles, the visible light (except purple) that passes through the filter appears almost unaltered, which is a great improvement upon the traditional high-optical-density red safety goggles that must be worn when working with purple lasers of similar intensity. Standard dark-red goggles filter all colors except red, which is also reduced, generally decreasing user visibility. We tested dark-red goggles for detecting red-marked BMSB with the laser, and found they performed poorly (data not presented).

To our knowledge, this study documents the first use of a powerful, handheld, near-UV laser to detect fluorescent pigments. This method greatly enhances effective detection distances and efficiency relative to previous methods, and should allow long-distance tracking of fluorescent-marked organisms even in dense tree canopies and aquatic environments. Use of a laser, as detailed here, is only possible with the specially designed goggles we described because conventional safety goggles would greatly interfere with visibility and detection. By substantially improving re-sight distances and making in-field detection more feasible, we expect that this non-destructive re-sighting technique will facilitate a great range of ecological studies, including observation of cryptic nocturnal behaviors and tracking the spatial movement of invasive and endangered species and other economically and ecologically important species.

### Warning

Lasers are extremely dangerous for eye exposure. NEVER use lasers without safety goggles. Use extreme caution when working with lasers. ALLWAYS adhere to safety guidelines provided by the laser manufacturer. The purchase of high-power lasers may require special permitting in some states.

## References

[1] Hagler JR, Jackson CG (2001) Methods for marking insects: current techniques and future prospects. Ann. Rev. Entomol. 46:511–543. 11112178 10.1146/annurev.ento.46.1.511

[2] Bryne DN, Rathman RJ, Orum TV, Palumbo JC (1996) Localized migration and dispersal by the sweet potato whitefly *Bemisia tabaci* . Oecologia 105:320–328. 28307104 10.1007/BF00328734

[3] Corbett A, Rosenhiem JA (1996) Quantifying movement of a minute parasitoid, *Anagrus epos* (Hymenoptera: Mymaridae), using fluorescent dust marking and recapture. Biol. Control 6:35–44. 8878740

[4] Fargo RJ, Laudenslayer WF Jr (1997) Use of night vision goggles, light tags, and fluorescent powder for measuring microhabitat use of nocturnal small animals. Transactions of the Western Section of the Wildlife Society 33:12–17.

[5] Foltan P, Konvicka M (2008) A new method for marking slugs by ultraviolet fluorescent dye. J. Mollus. Stud. 74:293–297.

[6] Furman BLS, Scheffers BR, Paszkowski CA (2011) The use of fluorescent powdered pigments as a tracking technique for snakes. Herp. Cons. Biol. 6:473–478.

[7] Knittle CE, Linz GM, Johns BE, Cummings JL, Davis JE Jr, Jaeger MM. (1987) Dispersal of male red-winged blackbirds from two spring roosts in central North America. J. Field Ornithol. 58:490–498.

[8] Polivka JB, (1949) The use of fluorescent pigments in a study of the flight of the Japanese beetle. J. Econ. Entomol. 42:818–821.

[9] Quay WB (1948) A technique for the automatic color marking of shrews. J. Mammal. 29:225–234.

[10] Kearns CA, Inouye DW (1993) Techniques for pollination biologists Univ Press of Colorado, Niwot, CO.

[11] Longland WS, Clements C (1995) Use of fluorescent pigments in studies of seed caching by rodents. J. Mammal. 76(4):1260–1266.

[12] Narisu J, Lockwood A, Schell SP (1999) A novel mark–recapture technique and its application to monitoring the direction and distance of local movements of rangeland grasshoppers (Orthoptera:Arcididae) in the context of pest management. Journal of Appl. Ecol. 36:604–617.

[13] Pal R (1947) Marking mosquitoes with fluorescent compounds and watching them by ultra-violet light. Nature 160:298–299. 20344640 10.1038/160298b0

[14] Reiter J, Curio E, Tacud B, Urbina H, Geronimo F (2006) Tracking bat-dispersed seeds using fluorescent pigment. Biotropica 38(1):64–68.

[15] Coviella CE, Garcia JF, Jeske DR, Redak RA, Luck RF (2006) Feasibility of tracking within-field movements of *Homalodisca coagulata* (Hemiptera:Cicadellidae) and estimating its densities using fluorescent dusts in mark–release–recapture experiments. J. Econ. Entomol. 99(4):1051–1057. 16937655 10.1603/0022-0493-99.4.1051

[16] Stern VM, Mueller A (1968) Techniques of marking insects with micronized fluorescent dust with especial emphasis on marking millions of *Lygus hesperus* for dispersal studies. J. Econ. Entomol. 61(5): 1232–1237.

[17] Warner KA, Bierzychudek P (2009) Does marking with fluorescent powders affect the survival or development of larval *Vanessa cardui*? Entomol. Exp. App. 131:320–324.

[18] Akey DH, Hayes JL, S. J. Fleischer [eds]. 1991. Use of elemental markers in the study of arthropod movement and trophic interaction. Supplement No. 14 of Southwest. Entomol. Southwestern Entomological Society. Dallas, TX. 87 p

[19] Fleischer SJ, Gaylor MJ, Hue NV (1988). Dispersal of *Lygus lineolaris* (Heteroptera: Miridae) adults through cotton following nursery host destruction. Environ. Entomol. 17(3): 533–541.

[20] Nagoshi RN, Fleischer SJ, Meagher RL (2009) Texas is the overwintering source of fall armyworm in central Pennsylvania: Implications for Migration into the northeastern United States. Environ. Entomol. 38 (6): 1546–1554. 20021748 10.1603/022.038.0605

[21] Nagoshi RN, Meagher RL, Hay-Roe M (2012) Inferring the annual migration patterns of fall armyworm (Lepidoptera: Noctuidae) in the United States from mitochondrial haplotypes. Ecol. Evol. 2(7): 1458–1467. doi: 10.1002/ece3.268 22957154 10.1002/ece3.268 PMC3434929

[22] Leskey TC, Hamilton GC, Nielsen AL, Polk DF, Rodriguez-Saona C, Bergh JC, et al (2012) Pest status of the brown marmorated stink bug, *Halyomorpha halys* in the USA. Outlooks on Pest Management 23(5): 218–226.

[23] Rice KB, Bergh CJ, Bergmann EJ, Biddinger DJ, Dieckhoff C, Dively G, et al (2014) Biology, ecology and management of brown marmorated stink bug (*Halyomorpha halys*). J. Integrated Pest Management. 5(3) 1–13.

[24] Neenan STV, Hodgson DJ, Tregenza T, Boothroyd D, Ellis CD (2014) The suitability of VIE tags to assess stock enhancement success in juvenile European lobsters (*Homarus gammarus*). Aquacult. Res. 1–11.

[25] Willis TJ, Babcock RC (1998) Retention and in situ detectability of visible implant fluorescent elastomer (VIFE) tags in *Pagrus auratus* (Sparidae). New Zeal. J. of Mar. Fresh. 32:247–254.

[26] McFarlane GA, Wydoski RS, Prince ED (1990) External tags and marks. Historical review of the development of external tags and marks. Amer. Fish. Soc. Symp. 7:9–29.

[27] Hein S, Poethke HJ, Hovestadt T (2005) Computer-generated null models as an approach to detect perceptual range in mark–re-sight studies—an example with grasshoppers. Ecol. Entomol. 30:225–233.

[28] Lemke A, von der Lippe M, Kowarik I (2009) New opportunities for an old method: using fluorescent colours to measure seed dispersal. J. Appl. Ecol. 46:1122–1128.

[29] O’Neal ME, Landis DA, Rothwell E, Kempel L, Reinhard D (2004) Tracking insects with harmonic radar: a case study. Am. Entomol. 50: 212–218.

[30] Yang M, Luiken G, Baranov E, Hoffman RM (2005) Facile whole-body imaging of internal fluorescent tumors in mice with an LED flashlight. Biotechniques. 39:170–2. 16116787 10.2144/05392BM02

[31] Kaiser PK, Boynton RM (1996) Human color vision Second edition Optical Society of America Washington, DC.

[32] Maxwell K, Johnson GN (2000) Chlorophyll fluorescence-a practical guide. J Exp Bot. 51(345):659–68. 10938857 10.1093/jxb/51.345.659

[33] Pandey JK, Gopal R (2011) Laser-induced chlorophyll fluorescence: a technique for detection of dimethoate effect on chlorophyll content and photosynthetic activity of wheat plant. J Fluoresc. 21(2):785–91. doi: 10.1007/s10895-010-0771-5 21128104 10.1007/s10895-010-0771-5

